# Research and Innovation for and with Adolescent Young Carers to Influence Policy and Practice—The European Union Funded “ME-WE” Project

**DOI:** 10.3390/ijerph19169932

**Published:** 2022-08-11

**Authors:** Elizabeth Hanson, Francesco Barbabella, Lennart Magnusson, Rosita Brolin, Miriam Svensson, Stecy Yghemonos, Valentina Hlebec, Irena Bolko, Licia Boccaletti, Giulia Casu, Renske Hoefman, Alice H. de Boer, Simone de Roos, Sara Santini, Marco Socci, Barbara D’Amen, Frans Van Zoest, Nynke de Jong, Henk Herman Nap, Yvonne de Jong, Tamara Bouwman, Feylyn Lewis, Tom Parkhouse, Agnes Leu, Daniel Phelps, Elena Guggiari, Vicky Morgan, Francesca Centola, Stephen Joseph, Saul Becker

**Affiliations:** 1 Department of Health and Caring Sciences, Linnaeus University, 39182 Kalmar, Sweden; 2 The Swedish Family Care Competence Centre (NKA), Strömgatan 13, 39232 Kalmar, Sweden; 3 Eurocarers, 14 Rue Père de Deken, 1040 Brussels, Belgium; 4 Faculty of Social Sciences, University of Ljubljana, Kardeljeva pl. 5, 1000 Ljubljana, Slovenia; 5 Anziani e Non Solo Società Cooperativa Sociale, Via Lenin 55, 41012 Carpi, Italy; 6 Department of Psychology, University of Bologna, Viale Berti Pichat 5, 40127 Bologna, Italy; 7 The Netherlands Institute for Social Research (SCP), Postbus 16164, 2500 The Hague, The Netherlands; 8 Centre for Socio-Economic Research on Aging, IRCCS INRCA-National Institute of Health and Science on Aging, Via Santa Margherita 5, 60124 Ancona, Italy; 9 Vilans–The National Centre of Expertise for Long-Term Care in The Netherlands, Churchilllaan 11, 3527 Utrecht, The Netherlands; 10 Vanderbilt University School of Nursing, Godchaux Hall 226, 461 21st Ave S, Nashville, TN 37240, USA; 11 School of Education and Social Work, University of Sussex, Falmer, Brighton BN1 9RG, UK; 12 Department Health, Kalaidos University of Applied Sciences, Gloriastrasse 18a, 8006 Zürich, Switzerland; 13 Medical Faculty, Institute for Biomedical Ethics, University of Basel, Bernoullistrasse 28, 4056 Basel, Switzerland; 14 Faculty of Health and Well-Being, University of Winchester, Winchester SO22 4NR, UK; 15 Carers Trust, 32–36 Loman Street, London SE1 OEH, UK; 16 Mental Health Europe, Rue de la Presse 4, 1000 Brussels, Belgium; 17 School of Education, University of Nottingham, University Park, Nottingham NG7 2RD, UK; 18 Faculty of Health and Education, Manchester Metropolitan University, Manchester M15 6BX, UK

**Keywords:** young carers, mental health, social exclusion, primary prevention

## Abstract

Young carers are children and adolescents who provide care to other family members or friends, taking over responsibilities that are usually associated with adulthood. There is emerging but still scarce knowledge worldwide about the phenomenon of young carers and the impact of a caring role on their health, social and personal development spheres. This paper provides an overview of the main results from the ME-WE project, which is the first European research and innovation project dedicated to adolescent young carers (AYCs) (15–17 years). The project methods relied on three main activities: (1) a systematization of knowledge (by means of a survey to AYCs, country case studies, Delphi study, literature review); (2) the co-design, implementation and evaluation of a primary prevention intervention addressing AYCs’ mental health (by means of Blended Learning Networks and a clinical trial in six European countries); (3) the implementation of knowledge translation actions for dissemination, awareness, advocacy and lobbying (by means of national and international stakeholder networks, as well as traditional and new media). Project results substantially contributed to a better understanding of AYCs’ conditions, needs and preferences, defined tailored support intervention (resilient to COVID-19 related restrictions), and significant improvements in national and European policies for AYCs.

## 1. Introduction

Generally, childhood is seen as a protected stage of life and one without major responsibilities, where adults are “in charge” and provide care, while children are primarily beneficiaries of care. According to the United Nations (UN) Convention on the Rights of the Child (1989), a child (any individual below the age of 18 years), is entitled to receive protection and care necessary for their wellbeing. However, for a number of children across Europe this is far from being the case as they provide care, often on a regular basis, to a family member, such as a parent/s, siblings, other relatives and/or friends in need of support (due to an illness, disability, addiction), and they find themselves assuming responsibilities that would be more fitting for an adult [1]. Statistics on the number of young carers (YCs) (<18 years) in Europe vary, but from the available national data, approximately 7–8% of all children carry out substantial amounts of caring [2,3,4,5,6].

Overall, in many European countries, with the notable exception of the United Kingdom (UK), YCs remain largely invisible to policy makers, public authorities and service providers alike. Thus, they are often unaware of the challenges faced by YCs and the possible measures to prevent or overcome them [7,8,9].

Whilst it is important to recognise the positive impacts of caring among children and young people, such as satisfaction from caring, and an increase in self-esteem, empathy and maturity, especially when they play the role of auxiliary carers within multiple generation families [10,11], without adequate support YCs often experience negative outcomes. In particular, the pressures associated with caring are considered a risk for YCs’ mental health and well-being, with approximately 50% of all YCs experiencing care-related stress and 40% experiencing mental health problems [12]. There are also associated health inequalities during the life course with YCs at greater risk of being hospitalised for mental-ill health and experiencing suicidal feelings during adulthood [13]. YCs are also at risk of social exclusion with higher absenteeism [6] and drop-out rates from secondary school (without full grades) and low employability later in life [14]. Further, the negative impact at an individual level can also involve long-term negative consequences for society as a whole. A Swedish study highlighted that the long-term extra-societal costs for children of parents with mental illness and alcohol or drug abuse are EUR 3.5 billion per year [13]. These risk factors combined with a general lack of awareness by professionals, policy makers and among YCs and families themselves [15] acted as the rationale for this project.

This paper provides an overview of the main results arising from the first pan-European research and innovation project dedicated to YCs whose aim was to change the “status quo” with regards to the situation of YCs in Europe. This is the “ME-WE project, “*Psychosocial support for promoting the mental health and well-being among adolescent young carers (AYCs) in Europe*” funded by the European Union (EU) within the Horizon 2020 programme (2018–2021) (https://me-we.eu, accessed on 12 July 2022). The key target group was a sub-group of YCs, i.e., adolescent young carers (AYCs) aged 15–17 years of age. The rationale for choosing this group was that they are experiencing an important transition phase in their lives, from adolescence to adulthood, where personal and social identity are being formed and completion of secondary education is a key factor for transition to adulthood and entry to the labour market and higher education [16]. There are of course other transition points for young carers (from primary to secondary school; from secondary school to college or university; from education to paid employment; from caring to independence/non-caring, etc.). The specific focus here on adolescent young carers aged 15–17 is important because (a) this is an under researched group/subset of the young carers population and we need to understand their specific experiences, caring roles and outcomes *during* this phase. It was recognised that AYCs are an under-studied group of YCs and, prior to the COVID-19 pandemic, there were relatively few studies focusing on AYCs’ mental health and well-being, and particularly from a primary prevention perspective [17]; (b) these AYCs are at a key transition phase to adulthood whilst still legally being regarded and treated as children; (c) as children, their needs and service responses would come under the auspices of child welfare legislation and children’s services/interventions, which requires greater understanding and policy/practice development. Further, most of the studies conducted so far are mainly at the national, regional or local level so that a cross-national comparison is lacking [4]. The ME-WE project involved six European countries, Sweden (SE), the Netherlands (NE), Italy (IT), Slovenia (SI), Switzerland (CH) and the UK, all with varying levels of awareness of and support for (A)YCs [15]. All project partners are members of Eurocarers, a European non-profit, umbrella organisation that advocates for the rights of carers, including YCs and AYCs, across Europe with the support of 75 national stakeholders (https://eurocarers.org, accessed on 12 July 2022). Thus, the project was based on a Eurocarers consortium, with research and carers organisations working together to address AYCs’ needs and preferences. The idea was to promote a greater awareness of AYCs among stakeholders at both national and European levels, including policy makers, health and social care services, schools, the non-profit sector, and others.

The overall project goal was to strengthen the resilience of AYCs in order to impact positively on their mental health and well-being and to mitigate the negative influence of psychosocial and environmental factors in their lives. In this overview article, the main project results and research and innovation methods will be summarised and discussed in relation to the three core project objectives, which were as follows:

(1) To systematise knowledge on AYCs by (a) identifying their profiles, needs and preferences, (b) analysing national policy, legal and service frameworks, and (c) reviewing good practices, social innovations and evidence (Work Packages, WPs, 1–3);
(2) To co-design, develop and test, together with AYCs, a framework of effective and multicomponent psychosocial interventions for primary prevention focused on improving their mental health and well-being, to be tailored to each country context (WPs 4–6);
(3) To carry out wide knowledge translation actions for dissemination, awareness promotion and advocacy (WP7), by spreading results among relevant stakeholders at the national, European and international levels.

**Figure 1:**
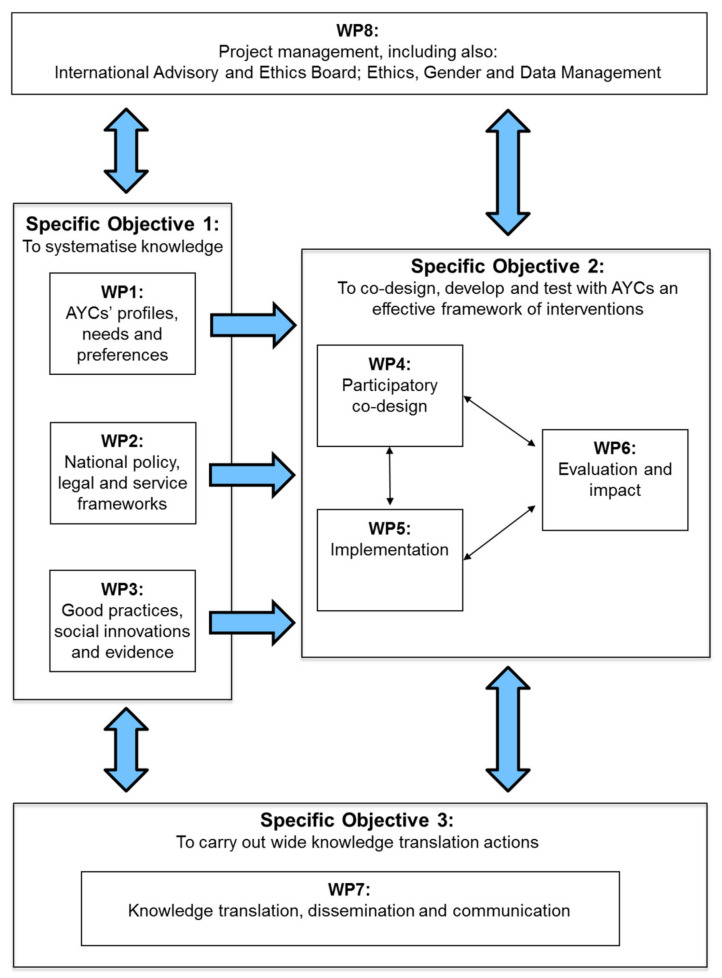
PERT chart of WPs and objectives in the ME-WE project.

Figure 1 shows the Work Packages (WPs), and their interrelations according to the three main research objectives. A work package in the context of an EU research project can be seen as a sub-project which, combined with other work package units, form the completed project.

## 2. Materials and Methods

The ME-WE project was designed as a mixed-methods study. Each of the main project objectives (systematisation of knowledge, primary prevention intervention, knowledge translation) had further specific aims, addressed by one or more WPs. We describe below the methods applied to achieve each project objective.

### 2.1. Objective 1: Systematisation of Knowledge on AYCs

In the first year of the project, research activities consisted of gathering available empirical and policy literature, in addition to collecting new qualitative and quantitative data and subsequently structuring and synthesising the data to highlight the “state of the art” in the area and to identify the current knowledge gaps that would help inform the next phase of the work (see Figure 1 above). More specifically, the initial phase consisted of the following research methods:

(1) A cross-national comparative study of the profiles, needs and preferences of AYCs using an online survey (conducted in WP1). The survey was based on a structured examination of the literature and previous relevant online surveys to identify key areas of inquiry and to enable appropriate survey questions to be developed. The survey included screening questions to determine whether a person was an AYC. The following series of questions was designed with the aim of capturing responses from AYCs who might not have previously considered or thought of themselves to be an AYC:
  - Q1. Do you have someone in your family with a health-related condition?
  - Q2. What type of health-related condition do these persons have?
  - Q3. Who are these persons (for example, parent[s], sibling[s], grandparent[s] and so on)?
  - Q4. Do you live with the family members who have a health-related condition?
  - Q5. Do you look after, help or support any of these family members with a health-related condition?

Respondents were also asked to report whether they “look after, help, or support” friends or other individuals close to them. Affirmative answers to either the “family” or “friends” support question classified a respondent as a carer. Their reported ages were used to determine if they could be classified as an AYC; only carers of the ages 15–17 years old were classified as AYCs.

Other questions in the survey consisted of:

- A series of demographic questions;
- Three instruments: The Multidimensional Assessment of Caring Activities (MACA) [18,19], The Positive and Negative Outcomes of Caring (PANOC) [18,19] and Kidscreen 10 Measure of Health-Related Quality of Life [20];
- Impacts on Education, Employment and Support section and an open-ended qualitative question: “If you’re looking after someone, what would help support you as a carer?” A further open-ended optional question: “If you are caring for an older person (aged 65 and over), what are the main difficulties you are facing?” was asked in Italy and in Slovenia respectively, two countries characterized by a familistic welfare system where it was expected that intergenerational caring was quite common. Further information on the specific analysis of AYCs of people aged 65 and over, mainly grandparents, is published elsewhere [17,21].

The English version of the online survey was then translated by each country partner into the following languages: Italian, Dutch, Slovene, Swedish, Swiss, German, Arabic and Dari, according to partners’ specific country contexts. Further information on the survey methods is published elsewhere [8,10]. Data were analysed using IBM SPSS Statistics and included descriptive data such as frequency, mean, and standard deviation. To study the impact of caring in a cross-country analysis, inferential tests were also performed: independent-samples *t*-tests, paired-samples *t*-tests, and Pearson’s correlation coefficient.

(2) A qualitative analysis of the development and implementation of policies, legislation and services addressing AYCs in the six partner countries (conducted in WP2). It consisted of the following:
  - A preliminary examination (web-based search) for policy responses to YCs;
  - Semi-structured interviews with experts in the field of AYCs and related legal provisions in all six partner countries (in total 25 interviews) to explore what specific legislation exists to protect YCs, how the legislation defines and constructs YCs, what other (non-specific) legislation can/or has been used in the context of YCs, how the procedures work in practice, their strengths and limitations, how the changes in policy and legislation were achieved and goals and hopes for the future;
  - Based on both these data sources, country case study analyses were carried out to provide a rich description of how YCs are supported and protected in each country, both by the law and its enactment and with a focus on identifying the limitations and how progress was achieved;
  - A cross-national synthesis of the country case study analyses was then conducted to compare the progress made in each of the partner countries which formed a report;
  - Former YCs gave their feedback on the draft version of the report [22].

Further information on the case study methods was published elsewhere [7,22];

(3) A systematic overview of successful strategies to improve AYCs’ mental health (conducted in WP3), which consisted of three sources of data as follows:
  - A Delphi study with 66 experts from the six partner countries and from Austria, Belgium, Ireland and Germany who were all working in the fields of young carers, alternatively in related fields (such as youth policy), if young-carer-specific policy was not available in the country. They included researchers and policy makers together with representatives from end user organisations and industry. The eligibility of the experts was cross-checked by the national investigator teams. The experts from the additional countries were included because of their expertise of European law and policy. Interviews were held in two rounds. Three main topics were selected for the open-ended questions in the first Delphi round:
    - Visibility and awareness raising of (A)YCs on a local, regional and national level;
    - Current strategies, interventions and/or programmes to identify support to (A)YCs, including pros and cons;
    - Future needs to support the well-being and health situation of (A)YCs;
  - A systematic literature review in which 2500 papers were reviewed and reduced to 40, focused on support for YCs, together with a general literature review and social media analysis;
  - A rating, ranking and consolidation task in which experts and YCs rated a selection of 39 interventions, programmes and methods identified in (a) and (b) according to criteria which included the influence of the support programme on mental health, education, resilience and transferability of the programme to an online platform/app.

The Delphi study consisted of two rounds. In both rounds, the interview data were first analysed by the national project teams using narrative analysis. The interviews were transcribed, and themes were identified, analysed and summarized on a national and international level. The results were presented in narrative form according to the main interview themes. A thematic analysis resembles the actual process and the results were presented in narrative form.

The Delphi study data were coded by three data coders using a code tree with an initial set of broad concepts offering flexibility to add regional or national themes using the qualitative data analysis software MAXQDA of VERBI GmbH. The content was analysed and discussed among national teams and subsequently with researchers from the additional countries, resulting in a summary discussion by the Netherlands team leading the Delphi Study. All interviews were labelled and manually coded and relevant quotes were selected that represented the underlying themes, i.e., exemplar quotes for the theme and (sub)themes (e.g., on visibility, future needs for funding or responsibility, training of professionals). These county-level data were aggregated and analysed, resulting in an overview of overall themes and country or cultural specific insights.

Simultaneously with the Delphi study, the academic literature on interventions to support YCs (below 18 years, including AYCs) and young adult carers (18–24 years) was reviewed. We also targeted young adult carers in order to have a complete overview of the available evidence on possible interventions and good practices across childhood and young age, which could have brought useful inputs and lessons also for our primary target group (AYCs). The systematic literature search was executed in the PubMed, Psychinfo and Embase databases for articles published about YCs. The inclusion criteria were YCs and young adult carers younger than 24 years, written in English or Dutch, and evaluating an intervention or support program, and the exclusion criteria were published before 2006 and older than 24 years. Two researchers selected the articles, making use of the software Rayyan. The review gave insights into the academic literature which could be used in the next phase of the rating, ranking and consolidation task. A general literature and social media analysis was also conducted. The social media monitoring tool Coosto was used and the platforms included were Twitter, Facebook, YouTube and Instagram.

Following completion of the Delphi study and literature reviews, 39 interventions, programmes and methods were included and deemed eligible for the ME-WE intervention. In the rating, ranking and consolidation task, all interventions were rated by YCs and experts. The criteria for basing the scores included the current status, openness, level of co-creation with YCs, influence on well-being or resilience and adaptability to a digital platform, which were deemed important in the expert interviews and BLN discussions. The reviewers used the Mean Opinion Score scale from 1 (low) to 5 (high). Most reviewers completed their ratings autonomously. The ratings led to a ranking of the interventions, scored on the different criteria.

Further information on the Delphi, literature review and ranking methods is published elsewhere [9,23].

### 2.2. Objective 2: Co-Design, Implementation and Evaluation of an Intervention Framework

#### 2.2.1. Co-Design Phase

The second project objective involved co-design and participatory research methods that ran throughout the project in WP4 (see Figure 1). To ensure that the voices of AYCs and key stakeholders were heard, national Blended Learning Networks (BLNs) [24] were established in all six partner countries and held approximately every 8–12 weeks. BLNs are heterogeneous communities of practice that, in the context of the project, consisted of AYCs and YCs, former AYCs and YCs, together with key stakeholders: school staff, youth workers, health and social care professionals, members of civil societies, together with ME-WE team members.

A discussion guide was developed by the project coordinator and circulated to the BLN facilitators, who often comprised two members of the ME-WE project team per country. One of the facilitators led the sessions and a second member acted as an assistant and, with members’ permission, made detailed minutes of the discussions that took place which were then sent to the project coordinator. A member of the coordinating team made a synthesis of the country minutes which were then fed back to the consortium and helped inform, for example, the ME-WE mobile app, intervention framework, the clinical trial and mixed-methods study.

An additional form of co-design was the setting up of local design user groups in Sweden, the Netherlands, Slovenia, Italy and Switzerland from January to October 2019, consisting of AYCs and a national team member and led by the coordinator, in order to carry out further development and initial testing of the ME-WE mobile app.

At a European and international level, the project coordinator regularly consulted with members of the Eurocarers Young Carers Working Group (n = 30 YCs, young adult carers and former YCs from 10 EU countries and Australia), initially at the project proposal stage and subsequently on approximately a six-monthly basis throughout the project period. It included two co-design workshop sessions which focused on members’ feedback and suggestions for the content and functionality of the ME-WE mobile app and the ME-WE intervention framework.

Further, members of the project’s external International Advisory and Ethics Board (IAEB), which met online on a bi-annual basis, included equal numbers of young carers (n = 4) and policy and research/academic representatives (n = 4). The ME-WE consortium received recommendations, guidance and comments from the IAEB during the entire project period. On a yearly basis, the IAEB reviewed and approved an Ethics Report with the periodical outcomes of the ethical assessment of project work.

The main results arising from the systematised knowledge (Phase 1), together with the core discussion outcomes and lessons learned from the BLN sessions in year 1, served to inform the ME-WE psychosocial intervention framework.

**Figure 2:**
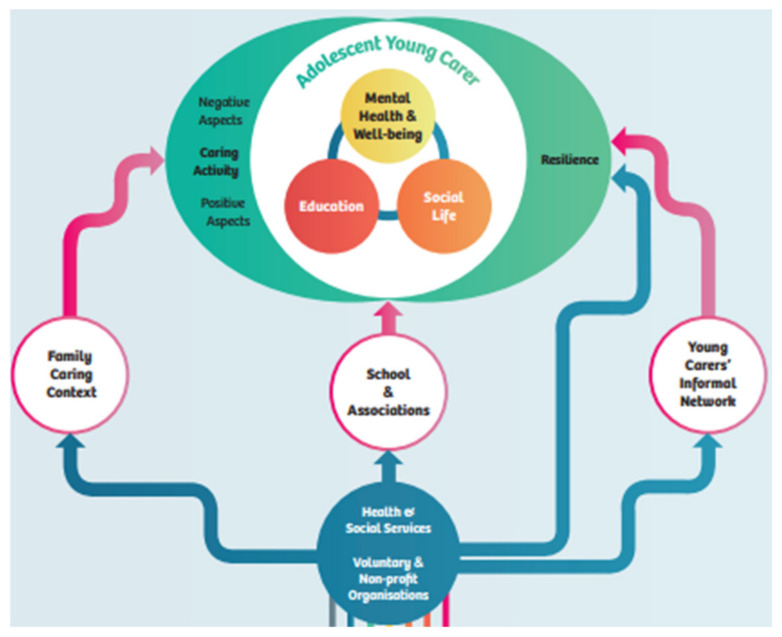
The ME-WE project conceptual framework.

The ME-WE psychosocial intervention framework was co-designed with YCs on the basis of the project conceptual framework (see Figure 2), which considers the psychosocial facets of AYCs’ lives and the social actors who may effectively support them.

The theoretical framework agreed on for developing the ME-WE intervention was the DNA-V Model (Discoverer, Noticer, Advisor and Values) [25]. The DNA-V model is grounded in an Acceptance and Commitment Therapy (ACT) evidence-based approach [26] and is suitable for working with adolescents to promote their well-being. The DNA-V model was deemed to be especially coherent with the objectives of the ME-WE project, i.e., to promote the mental health and well-being of AYCs who are in a critical transition period from adolescence to adulthood. The educational journey involved in the DNA-V model was intended to help AYCs in recognizing, accepting, and sharing the emotions aroused by their caring experiences, which are often held silent [27]. The DNA-V model was also seen to have the potential to help AYCs: (i) explore and expand their behavioural repertoires to develop new, effective ways of being in their caring role (if they wish to) and in social relationships and (ii) develop a flexible self-view and explore identities and future opportunities [28].

The general objectives of the ME-WE primary prevention support intervention targeting AYCs 15–17 years of age were as follows:

(1) Promoting good mental health and well-being and enhancing resilience among AYCs;
(2) Enabling AYCs to recognize and accept their internal experiences;
(3) Enabling AYCs to experience new or alternative behaviours and build strengths;
(4) Promoting AYCs’ sense of self-worth by developing self-awareness, self-knowledge and useful self-concepts, cultivating mindfulness;
(5) Enabling AYCs to build supportive social networks;
(6) Enabling AYCs to be flexible in facing life events and live according to their values.

The key findings from the systematised knowledge outlined above helped to inform the ME-WE intervention framework. The co-design of the intervention took place in several BLNs sessions in all six countries and results were compiled and synthesized by the coordinator. In summary, the ME-WE common intervention framework consists of seven weekly group sessions with home exercises in between sessions and a follow-up session at three months post-intervention, led by two trained facilitators.

**Figure 3:**
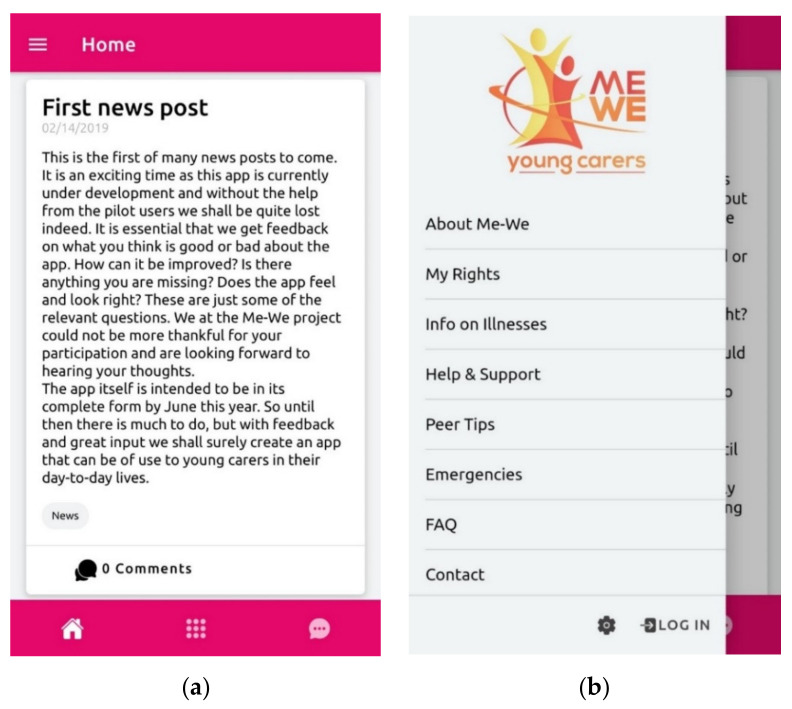

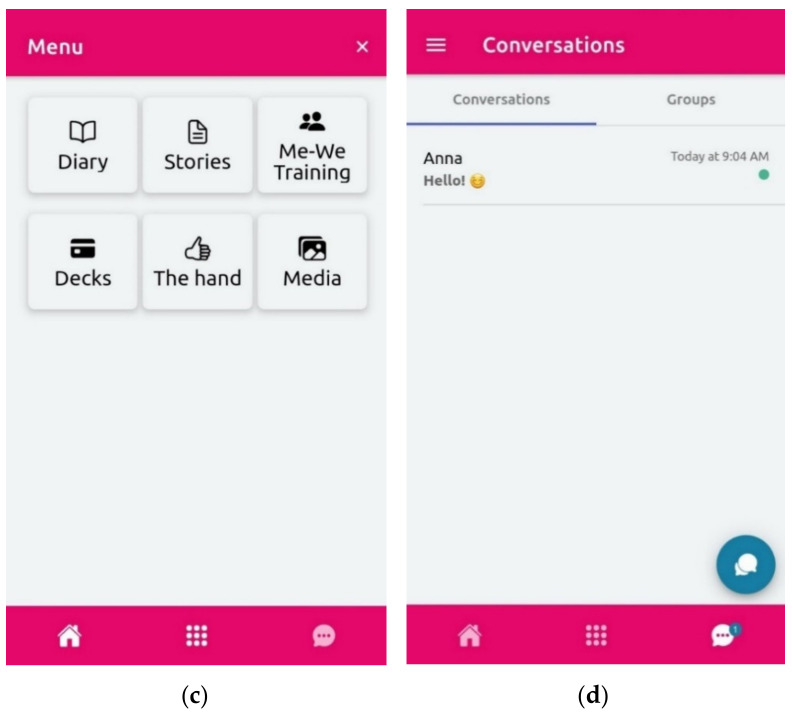
(**a**) ME-WE app: Homepage includes country-specific news and is the first page that appears when a user starts the app. (**b**) ME-WE app: Left menu includes pages with relevant information concerning YCs, a log out/log in button and settings where users can change their username, password, text size, etc. (**c**) ME-WE app: Launchpad menu, a navigation area where the Diary, Stories and the educational resources used in the ME-WE group sessions can be found. (**d**) ME-WE app: The chat where users can send text messages and pictures in either individual conversations or group conversations.

Furthermore, the technical specifications and core content for the ME-WE mobile app were co-designed initially in the BLN sessions and subsequently in the local user groups, where they were also initially tested in three partner countries. A software company contributed to the technical development of the app according to given specifications. The app was subsequently field tested in the context of the clinical trial study in Sweden, the Netherlands and Switzerland and as “stand alone” field testing sessions with AYCs in the UK, Italy and Slovenia, respectively. Figure 3a–d below highlight the key functions, “look and feel” and content of the mobile app which is currently publicly available on Google Play (https://play.google.com/store/apps/details?id=se.appbolaget.mewe&hl=en_US&gl=US, accessed on 12 July 2022) and App Store (https://apps.apple.com/se/app/me-we-young-carers/id1452257199?l=en, accessed on 12 July 2022).

#### 2.2.2. Implementation and Evaluation Phase

A randomised controlled trial protocol was developed for the original evaluation design [16] (see Table 1 ME-WE Clinical Trial Study summary overview). This initially involved a systematic review of quantitative outcome measures concerning mental health, education, social life, family relationships and other socio-economic dimensions. Chosen measurement instruments for AYCs were pretested on a convenience sample of proxy (student) respondents to establish their psychometric properties and check for any overlap and duly select the superior instruments (see Table 1). The selected instruments were then translated into national languages using the TRAPD translation strategy [29]. The self-report tools selected to assess primary and secondary outcomes are listed in Table 1.

Two different delivery methods were initially agreed on for the delivery of the ME-WE intervention: a fully face-to-face approach (adopted by Italy, Slovenia, and United Kingdom), and a blended approach that combined face-to-face and online sessions delivered via the ZOOM platform and a dedicated ME-WE mobile app (adopted by Sweden, Switzerland, and the Netherlands).

Due to the onset of the COVID-19 pandemic and ensuing restrictions in place in all six countries (March 2020), the overall intervention delivery and evaluation methodology were adapted. The project consortium in cooperation with the national BLN members and IAEB members switched to fully online deliveries of the ME-WE intervention in all six countries. This involved the use of ZOOM and Microsoft Teams video-conferencing systems in Slovenia, Italy and the UK and the ME-WE mobile app, supported with ZOOM in Sweden, the Netherlands and Switzerland. However, no changes in essence were introduced to the core intervention content. See Casu et al. [16] for a detailed description of the ME-WE intervention framework and country deliveries. A further delivery approach, arising from the cooperation established in the national BLN with a national NGO in Slovenia, included a two-week camp delivery (Summer 2020).

Concerning the evaluation methodology, the project consortium adapted it to a robust triangulation, mixed-methods design to gain additional process evaluation data that could provide a more in-depth understanding of the implementation and evaluation of the ME-WE intervention in each of the six specific country contexts. The additional research methods included an “ad hoc” online survey and focus group interviews (all six countries) and/or individual interviews (Slovenia, Italy and the UK) with a range of key stakeholders in the partner countries, (see Table 2 for more details). These were carried out during the period May/June-December 2020.

Formal ethical approval for the study (by the relevant national, regional or university ethics committee) (SE, NL, IT, SI, UK) or detailed ethical opinions (CH) were duly secured in accordance with national legislation and in keeping with EU Horizon 2020 ethical guidelines (https://ec.europa.eu/research/participants/docs/h2020-funding-guide/cross-cutting-issues/ethics_en.htm, accessed on 12 July 2022).

Required sample sizes were estimated taking into account randomization occurring at the cluster (i.e., school or geographical area) level for all countries except Sweden and Switzerland. Due to COVID-related difficulties in recruiting participants, Sweden and Switzerland launched national social media recruitment campaigns, which posed no threats of contamination between study arms and determined endeavoured randomization occurring at the participant level in these countries. Final minimum total sample size (compensated for 20% attrition) was 526 AYCs (80 AYCs in Italy, 76 in Slovenia, 112 in the Netherlands, 142 in the United Kingdom, and 58 AYCs in Sweden and Switzerland). For more details on sample size calculations, see Casu et al. [16].

A short screening interview (face to face or via telephone) was carried out by research team members to assess the eligibility of potential participants in each country (see Table 2). At this time, a plain language statement was given to potential participants that clearly outlined the purpose of the study and they were asked to provide written parental/guardian consent or self-consent (as appropriate according to national legislation) to participate in the study. Participants were informed that their participation was entirely voluntary and that they had the right to withdraw from the study at any time without giving a reason and without any adverse repercussions.

Regular monitoring of data collection procedures was carried out by the designated national clinical trial data manager in each of the six countries to ensure compliance with the study protocol. Further, at project level, approximately monthly to six weekly clinical trial manager meetings were held, chaired by the project’s Ethics, Gender and Data Manager.

To support the implementation and monitoring of the ME-WE intervention, national implementation teams (researchers and carers/carer representatives or equivalent within the project consortium) and transnational implementation teams (organised by clusters of countries sharing similar levels of awareness on AYCs) were established, with the goal being to ensure consistency between the research design and the practical implementation of the AYC support intervention at country site level. A key task involved connecting relevant stakeholders to the project from health and social care, education, youth work, sport and culture sectors working with vulnerable children and carers, for the purposes of the clinical trial implementation.

Following completion of the data collection, the quantitative data files were prepared and descriptive statistical analyses were conducted at both national and cross-national levels. Survey outcomes across groups were compared with regards to both study arm (intervention and control group) and delivery mode (face-to-face and blended approach with respective turn to online delivery due to the COVID-19 pandemic). Statistical analyses were completely based on individual participant-level data (see Hlebec et al. [38] for further details).

For the qualitative data analysis, each partner country analysed and summarised their national qualitative data using content analysis, in accordance with guidelines provided by Linnaeus University (LNU). The guidelines included a coding tree in which the survey’s main topics formed the key categories. Each country team inductively added codes to the coding tree, specific for their country context. Coding was performed using traditional hand-mapping, Microsoft Excel, or MAXQDA. Selected illustrative quotes were translated into English by the research teams in each country. The national summaries were then synthesised by the research teams at SCP and LNU and duly checked by the rest of the research teams to provide a cross-country summary of the main qualitative findings, highlighting similarities and differences across the partner countries. A condensed version of the key cross-country results is presented in the Results section below.

### 2.3. Objective 3: Implementation of Wide Knowledge Translation Actions

Wide knowledge translation actions (KTAs) ran concurrently throughout the project and were aimed at raising awareness of the project’s rationale and results among relevant stakeholders and to coordinate and carry out a number of targeted dissemination and outreach activities at the EU and national level, actively building on the combined use of the ME-WE partners’ networks and communication tools. These actions were initially designed to build on pre-existing collaborations between the Eurocarers network (which includes all ME-WE project partners) and EU/national decision-makers and stakeholders of relevance to the project’s intent. These collaborations were gradually enhanced and expanded, building on the learnings generated by the project regarding the specific situation and needs of YCs in Europe as well as potential solutions to address them. KTAs were therefore structured around a regular monitoring of and contribution to ongoing policy dialogues of added value to the project’s goals (e.g., youth, mental health, education, employment, human rights). This process not only served to identify knowledge gaps and barriers, but it also allowed the ME-WE partners to continuously adapt the knowledge generated so as to support our target audiences in their own objectives as well as foster the development of new policy initiatives on YCs at EU/national level.

### 2.4. Ethics, Gender and Data Management Monitoring

Project partners continuously monitored the compliance of research work with ethical principles, gender issues, data management duties and applicable legislation in each country. An Ethics, Gender and Data Management Framework (EGDMF) was developed as the key internal document for this purpose, which was progressively updated during the entire project duration.

The ME-WE consortium appointed a number of external experts to the project’s International Advisory and Ethics Board (IAEB). The IAEB was established at the beginning of the project for providing advice and recommendations on the strategy and implementation of the project, as well for supporting the dissemination. The ME-WE IAEB was composed of 3 experts from academia, one of whom was a gender specialist, together with a policy specialist and 4 expert adult YCs/former YCs.

Members of the IAEB met periodically (8 online meetings overall) for updates, discussion and advice on project progress. After every 12-month period, the IAEB performed a detailed ethics, gender and data management assessment and provided recommendations for the consortium, in order to assure the best quality and ethics compliance regarding the ME-WE research.

An Ethics, Gender and Data Manager (EGDM) was also designated as responsible for working closely with all partner country intervention sites and reporting regularly on matters related to ethics, gender and data management to the project Steering Committee.

The highest level of compliance with ethical and research standards was assured. All country interventions were approved by competent local ethics committees and complied with applicable data protection laws.

## 3. Results

The main ME-WE results are reported below according to each project core objective.

### 3.1. Systematisation of Knowledge

#### 3.1.1. Profiles, Needs and Preferences of Respondent AYCs

In total, 9437 individuals participated in the online survey across the six countries, and a total number of 2099 AYCs aged 15–17 years old were identified. Regarding country samples, Sweden had a sample of 702 AYCs which constituted the largest sample, followed by the UK (402). Table 3 provides an overview of the profiles of respondent AYCs across the six countries.

Responses from the online survey indicated that the AYCs who participated in the survey, across all six countries, are taking on significant caring responsibilities for their family members and friends, with a number of reported ill mental and physical health effects. The emerging trends indicate that AYCs are often girls providing care for her mother with a physical disability. The exceptions are in Italy, where our data show that grandparents and specifically grandmothers possess the predominance of care needs. This may reflect the lack of formal community care programmes for their aging population and the preference for care to take place within the family home (see Santini et al. [10], Santini et al. [21] and D’Amen et al. [17] for more details). Further, according to our data, significant care is provided by siblings in Sweden (21% sister; 19% brother), the UK (22% sister; 25% brother), and the Netherlands (15% sister; 29% brother) (see Table 4). In total, 1444 AYCs indicated that they care for a family member, whilst 1121 reported that they care for a close friend. Further, 466 (22%) of AYCs who participated in our survey are dual carers, providing care both to a family member(s) and a close friend(s).

When compared to their non-caring peers, AYC respondents perform greater amounts of caring activities in the home (namely domestic activities, household management, financial and practical management, personal care, emotional care, and sibling care), as measured by the MACA questionnaire [18,19]. This effect is significant across each of the six countries (see Table 5, [8]). Overall, the female AYCs (M = 13.07, SD = 5.70) in our survey reported a greater amount of care activities compared to male AYCs (M = 11.24, SD = 5.16), *t*(3210.93) = 6.80, *p* < 0.001, *d* = 0.34, however the statistical significance varies between countries (see Table 6).

A total of 82.1% of respondent AYCs were in education at the time of completing the survey. Difficulties in school because of caring varied across the six countries, with high rates of negative school performance (37%) and bullying (36%) reported in the UK sample compared to the other partner countries. Further, the highest rates of reported mental health problems that were related to caring responsibilities were also among the UK sample (57%), followed by Switzerland (34%), Sweden (26%), and Italy (19%), Slovenia (15%) and the Netherlands (12%).

Of particular note are the relatively high rates of severe mental health impacts due to caring reported by respondent AYCs in all six countries, both with regards to reporting self-harming thoughts (UK, 28%, 18% CH, 11% NL, 11% SE, 9% IT and 7% SI) and harm to others (12% UK, 7% IT, 5% CH, SI, and SE and 3% NL).

Table 7 shows the proportion of AYCs in our survey who indicated that they had various forms of support in connection to their caring roles. Around a third of the AYCs’ families received government assistance (34%), though the proportion varied between the countries. For example, 65% of the AYCs in the UK indicated that their family received government assistance, whilst this proportion was just 22% in Slovenia. In the UK and Sweden, a higher percentage of AYCs reported that they receive support because they are a carer (45.8% and 41.8% respectively; see Table 7); the recruitment strategy of both countries involving carer support organisations likely influenced this finding. There was greater variability between countries when the AYCs reported whether their school was aware of their caring role: the UK had the highest percentage of awareness (58.6%), followed by Sweden (31.8%) and the Netherlands (30.8%); Slovenia (14.2%), Italy (10.8%), and Switzerland (9.1%) had the lowest reported percentage. Perhaps indicating that the AYCs have a considerable reliance on informal support from friends than school or employers, there were higher percentages across all six countries when asked if they have a friend aware of their caring role (see Table 7) (see Lewis et al. [8] for further details of the online survey results and analyses).

The above study data produced in the first year of the project were subsequently taken up by the project consortium and stakeholders and used in wide knowledge translation actions as highlighted in Section 3.3 of the results below.

#### 3.1.2. National Policy, Service and Legal Frameworks

The cross-national synthesis of the national case studies highlighted that it is only the UK which has legislation that specifically recognises children’s caring roles (Children and Families Act 2014 [39], the Care Act 2014 [40], Carers Act in Scotland [41], and the Social Services and Well-being Act in Wales [42]) which reflects its position as advanced according to Leu and Becker’s [15] classification system. Sweden, in its intermediate position, legally recognises the needs of children who have parents with a somatic illness, mental-ill health, disability and/or substance abuse or a parent/s who dies unexpectedly, within a paragraph in the Swedish health care act (Hälso och sjukvårdslagen, 2017:30, 5 kap. 7§ [43]). Overall, across the countries it was found that the recognition of children’s caring role is reliant upon “non-specific” legislation such as education, health and social care legislation, safeguarding and child protection legislation and family legislation [7]. When country and EU experts were asked how changes in legislation and policy were achieved, there was consensus that the active and sustained engagement of the following stakeholders was of central importance: ministries, family associations, private and state organisations and last but by no means least, young people and carers. Key drivers included other European legislation/policy (e.g., the Family Code in Slovenia that came into force after analysis of other European legislation, especially the German one) and ratification of the UN Convention on the Rights of the Child.

Experts also identified several key steps which they deemed necessary to progress policy and legislation, namely:

(1) Addressing key dilemmas: firstly, whether it is acceptable to have children in a caring role? Secondly, is specific legislation/policy necessary to protect and support AYCs?
(2) Further awareness and recognition of AYCs is needed. To this end, further national research work and a common definition of AYCs is required;
(3) AYCs should be recognised as an important target group for policy makers and to bring this about the emphasis should be upon prevention and early interventions. Furthermore, existing legislation and policy should be extended to usefully include AYCs;
(4) Involving YCs and AYCs and a range of key stakeholders in the process of campaigning for and in forming new directions in policy and practice.

See Leu et al. [7] for more detailed results and analyses.

The case study analysis work was closely linked to the work in Section 3.1.3 below.

#### 3.1.3. Review of Good Practices, Social Innovations and Evidence

Similarly to the main case study findings, both the Delphi study [9] and literature review findings confirmed the lack of visibility and awareness of the issue of YCs, yet acknowledged the differences between countries and regions. There were a number of strategies/approaches identified from the synthesized data to promote AYCs’ mental health and inform the subsequent intervention framework which can be summarised as follows:

(1) Early identification of AYCs;
(2) An approach that supports YCs to build trustworthy relationships and facilitate being in a constant dialogue with friends (for peer support) and family and/or professionals which is also important to prevent loneliness and enhance coping;
(3) An approach that supports respite and could promote and share activities for respite and promote online and actual offline contacts;
(4) An approach that does not solely benefit the care recipient, carer or professional, but from which all parties involved benefit, for example a more family-centred approach;
(5) An app can provide (indirect) access to professionals or to people whom are prior YCs who can provide both advice and emotional support and encouragement to YCs;
(6) Online support that is time, place and culture independent;
(7) As for point 4 in Section 3.1.2 above, co-creation of the intervention with YCs is essential.

All three sources of data (Section 3.1.1, Section 3.1.2 and Section 3.1.3 above) provided an in-depth “state of the art” concerning legislation, policy and supports for AYCs in Europe and helped identify the key steps needed to move research, policy and practice forward. This systematised knowledge was used by the project consortium to help inform the ME-WE psychosocial intervention (see Section 3.2 below).

### 3.2. Co-Design, Implementation and Evaluation of the Intervention Framework

#### 3.2.1. Co-Design: National BLNs: Members and Sessions

In total, 82 BLN sessions took place in the six countries in the project (22 in Sweden; 14 in Switzerland; 13 in Italy; 18 in the Netherlands; 9 in Slovenia; 6 in the UK). The national BLNs consisted of: in year 1, 36 AYCs/YACs/former YCs and 56 other stakeholders; in year 2, 40 AYCs/YACs/former YCs and 124 other stakeholders; in years 3 and 4, 18 AYCs/YACs/former YCs and 91 other stakeholders. See Table 8 for an overview of the categories of stakeholders who participated in the six national BLNs.

Table 9 provides an overview of the session discussion topics and key themes and/or outcomes.

The BLNs were self-evaluated by the members at the end of Year 1 and the main benefits included the exchange of YCs’ and professionals’ experiences, which led to new insights and increased awareness. Several common challenges identified by members were difficulties in finding meeting times that were suitable for both YCs and professionals, and difficulties in recruiting YCs (SE, CH) or professionals (NL) to the national BLNs. Overall, the BLNs acted as the main source of involvement for YCs and other key stakeholders across the six countries on a continuous basis throughout the project.

#### 3.2.2. Implementation and Evaluation of the Intervention Framework

A total of over 220 partner organisations and stakeholders were involved in the recruitment of AYCs to the clinical trial study and included BLN members and additional interested stakeholders from various sectors working with vulnerable children and carers (as specified in the Methods section above).

In total, 45 ME-WE intervention groups were carried out throughout the implementation period (October 2019–November 2020). Ten groups were carried out according to the original delivery methods of face to face in UK, IT and SI and blended in SE, NL and CH. Following the COVID-19 outbreak, all delivery approaches switched to being fully online and in total 35 groups were delivered via ZOOM/Microsoft Teams (UK, IT, SI) or via the ME-WE mobile app and ZOOM (SE, NL, CH). In general, groups consisted of between 2 and 9 AYCs.

A total of 478 AYCs were recruited to the clinical trial study and after screen failures and withdrawals 260 AYCs were formally included in the study. All countries enabled participants who were interested in participating yet did not meet the inclusion criteria (and no alternatives for support were available to them) to attend the ME-WE groups as compassionate cases. The number of compassionate cases increased during the COVID-19 pandemic and the total number was 52. Following the exclusion of compassionate cases in the study itself and dropouts, the final sample size reached was 110 AYCs in the intervention group and 107 in the control group in the six countries.

#### Key Evaluation Results

A summary of the main evaluation results is presented here (please note that detailed evaluation results of the clinical trial study will shortly be published separately by Hlebec et al. [38]). Analysis is based on data collected in five countries, without Switzerland (due to the low country sample size). Our final sample size (for the five countries) included 107 participants in the intervention group and 106 participants in the control group. Considering the delivery mode, four groups were analysed: the intervention group with face-to-face delivery approach (n = 75), the control group with face-to-face delivery approach (n = 90), the intervention group with blended delivery approach (n = 32), and the control group with blended delivery approach (n = 16). However, the number of AYCs participating in the intervention is in fact larger than is reflected through the final sample size. As outlined above, based on ethical and compassionate grounds, some AYCs were also offered to take part in the intervention, but they were not included in the evaluation analysis due to not meeting the inclusion criteria. Furthermore, it should be noted that results presented include all data collected, regardless of virtualization of the intervention following the COVID-19 pandemic. The project research team considers that the two original modes of delivery have been duly preserved.

The mean age of participants was 16.36 years (SD = 0.87), with 79.2% female participants. The share of participants with migrant background varied from 0% to 40.0% per study arm per country.

#### AYCs’ Mental Health, Well-Being, Personal Confidence and Cognitive Functioning

The results are inconclusive in terms of confirming our hypothesis, where greater improvements in psychological flexibility, mindfulness, resilience, subjective mental health and quality of life as well as in perceived emotional impact of caring and social support would be found in the intervention group compared to the wait-list control. However, we can observe slight improvements in AYCs’ resilience, mindfulness skills, mental and general health, although we cannot statistically confirm all of these improvements.

Results of the post-intervention self-assessment (for details see Table 2) indicate that almost all participants enjoyed most of the activities regardless of the delivery approach and they believed that the intervention had taught them useful things and that it was worth going to. Most of the participants also reported that the intervention made them feel good about themselves as well as about their family. Approximately half of the participants thought that the person they care for was better off because they had participated in the intervention. Positive changes around handling stressful thoughts and feelings in a better way were mentioned by participant AYCs in most countries. Quite common was also the ability to be more forgiving and kinder to oneself and/or to take better care of oneself.

#### AYCs’ Education and Employability

The ME-WE intervention positively impacted on the educational and employment opportunities for AYCs under study. Participants in both intervention groups reported being more able to do their homework and they found it less difficult to do well in school during the course of the intervention. In addition, process evaluation results also indicate that the intervention could have had some positive impact on school attendance and performance.

#### Impact of COVID-19 on AYCs

The impact of the pandemic on AYCs and their lives was assessed with additional open-ended questions inserted in the evaluation questionnaires (see Table 2). Below we present insights from the qualitative analysis of these open-ended questions. It is interesting to note that AYCs reported several positive changes as a result of the pandemic. Namely, due to online schooling and not having to travel to school, some AYCs expressed having more time for themselves, for favourite activities or for self-reflection, as well as having more time with their family. Among the negative responses, social isolation or not being able to meet up with friends were the most commonly reported drawbacks. This, as well as an increased level of caring responsibility, the feeling of being left alone with the care recipient (co-resident AYCs), and increased worry for the care recipient when not being allowed to see her/him (non-resident AYCs), affected well-being. Other issues mentioned at T1 (baseline) were more frequent conflicts in the family, which in turn could have affected their mental health with depression, feelings of loneliness, anxiety and self-injury being named. Furthermore, worry about school, fear of transferring COVID to loved ones, loss of relatives or postponed treatment/investigations were also mentioned. At T1, more respondents from Italy, the Netherlands and Slovenia reported positive changes rather than negative changes, while the vast majority of the AYCs from Sweden and the UK reported negative changes. At T2 (post-intervention), the negative aspects seemed to be more prevalent in all partner countries, although new insights and improved relationships were also reported by some AYCs.

Although the pandemic influenced the AYCs’ overall well-being and mental health, and they prevailed at T2, the pattern was not always so clear-cut, highlighting the complexity of the impact of the pandemic on AYCs’ daily lives. Results however should be treated with caution as they likely reflect specific country situations and restriction measures that were in place at the time of assessment. Unfortunately, it was not feasible for the ME-WE research team to clearly identify the possible pandemic effect on our study outcomes.

#### Testing of the ME-WE Mobile App by AYCs

The mobile app was tested in some countries and ad hoc questions were asked to participants in order to understand their satisfaction and app usability. Main results from respondents in Sweden and the Netherlands (n = 31) pointed out a sufficient satisfaction with the app at baseline, which slightly decreased over time (probably due to bugs and technical problems faced during the study period). AYCs were mostly satisfied with the appearance of the app and the group exercises, whereas they were mostly dissatisfied with the usability and the diary feature. The majority of the AYCs stated that the combination of the app and online meetings (via Zoom) worked well for most or at least half of the time.

In Sweden, most of the AYCs stated that the app (especially stories and information pages) had been helpful and supportive, whereas most of the AYCs in the Netherlands stated that the app had not. Qualitative feedback collected from AYCs clearly indicated the occurrence of technical issues during the implementation (malfunctioning, unable to access the app etc.) which negatively affected the participants’ experiences.

#### ME-WE Stakeholder Core Findings

In all six countries, additional process evaluation data were gathered through focus group and individual interviews with ME-WE stakeholders, together with an online survey regarding stakeholders’ views and experiences from the ME-WE clinical trial field study work (see Methods section above). In total, 81 stakeholders participated in the individual interviews and focus group interviews across the six countries and a total of 112 stakeholders from across the six partner countries completed an online survey. A summary of the main findings is reported below.

#### Views and Experiences of the ME-WE Intervention

Stakeholders in all countries reported positive aspects of the ME-WE intervention as highlighted below:

- The theory and relevance of the DNA-V model (NL, IT, SE, UK), for example highlighting strengths and values in the lives of AYCs;
- Development and implementation of a targeted support intervention for AYCs in the ME-WE project (NL, CH);
- Beneficial effect on AYCs (NL, UK), because they felt seen (IT), experienced peer support (NL) and were able to learn and use new tools in practice (NL, UK, SE), and also to learn to handle emerging difficult situations due to the restrictions during the pandemic (UK);
- Positive effect of the ME-WE intervention on facilitators: acquiring new skills/knowledge that can also be used in their future work, also among other groups (UK, SI, SE), having a positive impact on personal life;
- Research assistant/facilitator (NL), and deriving a positive feeling on delivering the ME-WE groups (NL, SE);
- Support provided by the ME-WE team (IT).

Areas for further development of the ME-WE intervention mentioned by stakeholders included the following:

- Broadening the target group of the ME-WE training (also 14 year old AYCs, and adolescents in general);
- More involvement of staff who already have a relationship with and built trust with young people, e.g., school nurses;
- More involvement of (multi-disciplinary) professionals to increase awareness, such as awareness programmes in schools, school nurses;
- A systems-/family-based approach to support AYCs;
- Ironing out the technical issues of the ME-WE app;
- Enabling AYCs to participate by facilitating a safe place during online sessions;
- A continuation plan for after the ME-WE intervention.

#### Awareness of AYCs

Many of the ME-WE stakeholders in the focus group interviews explained how they had gained an increased awareness of the situation of AYCs as a direct result of their involvement in the project. In contrast, however, the stakeholder survey findings indicated that the relatively low levels of awareness of AYCs among decision makers and practitioners (education, health and social care sectors) in general, especially during the initial project period, in most partner countries, acted as a barrier to recruitment of AYCs. Stakeholders admitted that the recruitment process posed a challenge in the ME-WE project that was further exacerbated and compounded by the COVID-19 pandemic. They noted a variety of contributory factors, apart from a low awareness and the COVID-19 pandemic, that may also have contributed to the recruitment difficulties, such as lack of self-awareness among AYCs themselves, not wanting to identify as an AYC, the motivation of young people and the risk of stigmatization. A full consideration of the recruitment issues in the project is provided by Barbabella et al. [44].

### 3.3. Knowledge Translation Actions (KTAs)

The overall results from the wide knowledge translation actions can be summarised within the three key types of KTAs, namely awareness raising, engagement, and development of policies and practices. Table 10 below provides an overview of the main methods for knowledge translation actions employed in the project, the target audiences and main outputs and results. Throughout the project, more than 8000 stakeholders were actively engaged via bilateral meetings/networking activities, from different fields: students; school professionals, health professionals; policymakers; social workers; youth services; researchers; NGOs; media; young people and YCs, YACs or former young carers.

The ambition of the ME-WE project was to contribute to the identification and support of YCs on the basis of national and European policies and practices shaped with them (i.e., co-produced). The consortium recognised that research and evidence-based awareness raising that actively engages with target audiences are key drivers of change in both the policy and practice arenas. The research findings were therefore continuously conveyed to relevant stakeholders and used as evidence to inform needed change, throughout the project. These were communicated in various ways and forms in order to reach all relevant stakeholders, including via accessible, non-scientific publications written in layperson’s terms (see Table 10 below).

Moreover, project partners organised/participated in numerous events to raise awareness on YCs and share the project findings, such as the 3rd International Young Carers Conference (https://eurocarers.org/2021-IYCC/, accessed on 12 July 2022), which hosted the Final Conference of the ME-WE Project (online, 3–6 May 2021) and brought together over 300 participants (carers organisations, researchers, practitioners, young carers and decision makers) from 17 European countries, as well as Australia, Canada, Japan and the United States of America.

Traditional and social media were also used to shed light on YCs and on the ME-WE project’s outputs among the general public. Achievable and tailor-made country sustainability plans were developed, in order to ensure that the ME-WE impact continues after the end of the project.

Table 11 shows the main EU policy developments and the contribution by the ME-WE project through advocacy and lobbying actions, led by the project partner, Eurocarers.

From the above summary KTAs, as well as the impact at EU level, it can also be seen that the ME-WE project has helped to increase awareness about YCs and the challenges they face among professionals, policy makers and YCs themselves in all the project countries. This is evidenced by the variety of advocacy and lobbying actions that were carried out at the national level (see Table 12). ME-WE also helped to bring about changes in practices, as the project enabled involved professionals to be more attentive to any signals of a caring role; to offer them support or to refer them to support services available at local level; and to *talk about* but also *talk with* AYCs. Some ME-WE facilitators in the project noticed that their views on AYCs changed with more recognition of the other roles young people with caring tasks have instead of merely focusing on their role as an AYC. Partnership working was created to support and identify YCs (e.g., between schools and care support centres or between social services, health care, youth centres and schools). Table 12 below provides concrete examples of the contributions of the ME-WE project to national policy and practice.

## 4. Discussion

We discuss the main ME-WE results and outcomes firstly in terms of the significance of the overall project and secondly in terms of its contributions to research and innovation. Further, we highlight the strengths of the project, especially in terms of the sustained involvement of YCs and the project’s direct impacts on practice and policy. Subsequently, we acknowledge the limitations of the project and highlight the novel situation of the COVID-19 pandemic. Finally, we provide key recommendations to policy makers, decision makers and service providers who work with or who come into contact with young carers.

### 4.1. Advances in Knowledge, Research and Innovation

We consider that the project is significant in terms of its ambition, scale and innovation. It is the first cross-national study in six European countries of AYCs aged 15–17, which uses the same (and standardised) instruments to enable cross-country comparisons and analysis at the aggregate level. Whilst there is research being carried out in a number of countries globally, nevertheless there is a dearth of cross-national research that has been conducted so far [4]. The project includes major data and experiences from six European countries, making comparisons between countries feasible. Second, it focuses on the under-studied phenomenon of AYCs and their mental health and well-being, leading to new insights into the situation of AYCs and ways to prevent or mitigate the negative impact of caring on their lives as they transition to adulthood. Further, it acts as the first larger-scale programme to endeavour to demonstrate the impact of a primary prevention psychosocial intervention focusing on improving the resilience of AYCs. Previous intervention studies targeting AYCs and YCs have largely been small-scale, local, qualitative studies [9].

The project provides direct contributions to research in the field of AYCs, YCs and caring. There are already several within-country publications recently made available on AYCs, YCs and young adult carers (see, for example, in France [61], the Netherlands [62,63], Sweden [64,65], Switzerland [5,66], United Kingdom [14,67]), but this is the first study using a cross-country perspective with empirical data of AYCs themselves, for example, in the work with the WP1 online survey and the findings of the severe mental health impacts of caring on AYCs. Furthermore, it presents new knowledge of the initial impact of the COVID-19 pandemic on AYCs in the six partner countries. In addition, ME-WE brought together, synthesised and systematised existing knowledge and experiences, together with newly collected data that were then consolidated into scientific reports and publications for the research community. At the same time, the consolidated knowledge was also made available in more accessible formats so that it could be readily taken up and used by policy makers, decision makers and practitioners working in health and social care services and in schools. Further, the project provides details of a promising, primary prevention psychosocial intervention, particularly with regards to its impact on school attendance and performance. The evaluation design includes a mixed-methods approach and a suite of evaluation instruments that can be used and applied by interested researchers in the field.

A major critical success factor and innovative aspect of the project that was identified and confirmed by the project stakeholders and external International Advisory and Ethics Board members (IAEB) is the sustained and active involvement of YCs and relevant stakeholders throughout the project. In this way, the YCs’ involvement was seen to move beyond tokenistic gestures towards more genuine and meaningful involvement throughout the research and innovation process, as evidenced in the partnership working in the national Blended Learning Networks (BLNs). For example, in the co-design work with the intervention and especially the mobile app and also by acting as ambassadors in increasing awareness at diverse campaigns and by sharing knowledge as experts by experience. Thus, the BLNs proved to be an innovative method for participatory co-design, enabling the voices of YCs and those of key stakeholder groups to be heard and acted upon in a systematic way. In addition to the BLNs, YCs were valued and viewed as experts by experience via their equal membership of the IAEB alongside researcher and policy maker members and further, via their involvement at the online 3rd International Young Carers Conference and sessions at the European Parliament, together with dedicated sessions of the Eurocarers Young Carers Working Group. As a result, the phrase: “nothing about us, without us” became central and researchers and policy makers became aware of their own blind spots.

In addition to the innovative, participatory research method of BLNs, the other major innovative elements of the project include the co-design of the dedicated ME-WE YCs’ mobile app, which is now publicly available, free of charge, on App Stores and Google Play. The app can be used both as a tool in the ME-WE intervention and as a separate support for AYCs and YCs. Despite some participant AYCs indicating the occurrence of technical issues during the implementation phase, which to some extent negatively affected their experience of the app, it can nevertheless be argued that the app has potential to be a supportive tool for AYCs and YCs.

### 4.2. Advances in Awareness, Response and Policy

It can be seen that the availability of systematised knowledge and research evidence in the field of YCs in general, and AYCs in particular, together with a range of awareness- raising activities in all six countries led to making direct impacts on practice, especially, but not exclusively, in the partner countries of Slovenia, the Netherlands and Sweden, respectively (see also Table 12 above). This is largely due to a range of research activities, combined with the extensive awareness-raising activities carried out by the national project teams together with their stakeholders.

It can also be acknowledged that the mix of partners within the project consortium, consisting of researchers in the field of YCs and carers in general, working in partnership with representatives from carers organisations and the Eurocarers secretariat based in Brussels, enabled the co-creation of robust and reliable research evidence and, at the same time, facilitated a diverse range of knowledge translation actions and outputs and extensive, diverse awareness-raising activities to take place throughout the project and beyond. Taken together, this provided the impetus to lead to real changes in policy/policy developments concerning the issue of YCs, as well as gender equality and employment, at both the EU and national/regional level (see Table 11 above). Importantly, EU policy developments are expected to have repercussions in the long run and in all EU member states (and neighbouring countries), i.e., not only in the countries addressed by the ME-WE project.

Further, due to the blend of partners within the ME-WE consortium, there is also a stronger likelihood for the impact of the project to be sustained beyond the lifetime of the project, as evidenced by the concrete ongoing practice development work and policy achievements.

### 4.3. Project Limitations and Challenges

Whilst the project is significant, it does nevertheless have limitations which are mainly related to the issue of recruitment of AYCs to the clinical trial study. This represented a major challenge even prior to the COVID-19 pandemic, but it was further exacerbated by the pandemic which broke out as recruitment in schools and via service providers was in progress in all six countries, resulting in lower numbers of participants than expected. The recruitment challenges pose the dilemma of which should ideally come first, i.e., awareness raising and education activities with those professionals working with or coming into contact with YCs, or research work. We would argue that awareness raising directed at key stakeholder groups without solid research evidence is not likely to prove effective, hence our strategy to systematise the knowledge on AYCs in the first year of the project which was then made into accessible outputs directed at practitioners, decision makers and policy makers, both at national and EU level to help with awareness raising.

We acknowledge that the low number of AYCs recruited to the clinical study make it unfeasible to offer entirely clear statements about whether and how the ME-WE programme increased psychological flexibility, mindfulness, resilience, subjective mental health and quality of life. Nevertheless, in light of the overall mixed-methods approach, and in particular the process evaluation data, the potential of the ME-WE programme to strengthen AYCs’ resilience, contribute positively to their mental health and well-being and to mitigate the negative impact of psychosocial and environmental factors has been highlighted in the clinical trial study.

The project call requirements, together with a relatively short project timeframe for recruitment, our chosen age range (15–17 years) and the taboo and stigma around self-identification as a YC, all combined to create significant challenges with recruitment. A full consideration of the recruitment issues in the project is provided by Barbabella et al. [44]. Drawing on the lessons learned in the ME-WE project, we would argue for a mixed-methods approach to the evaluation of the ME-WE programme and to possibly include YCs also at an earlier age within the ME-WE programme as data from the online survey identified that some AYCs reported caring for a parent/someone close to them “for as long as they could remember”.

As a significant amount of recruitment and intervention delivery took place during the COVID-19 pandemic, the results are unique due to this extraordinary period of time. In addition to exacerbating the recruitment difficulties, the pandemic and ensuing restrictions in all the partner countries meant that the original face to face delivery of the ME-WE groups was not feasible. Nevertheless, the need to redesign the ME-WE intervention mode of delivery from face to face to fully online created an unexpected opportunity as it acted as a pilot study of an online mode of delivery. It can be argued that the online version of the ME-WE intervention offers a new supportive programme for AYCs living in hard-to-reach areas, those AYCs living in regions without adequate support available, or those AYCs who are not enrolled in school or education, who are able to have access to the ME-WE intervention because of its online delivery. Thus, greater access to the ME-WE programme is now assured through the online mode of delivery, allowing AYCs who are even more considerably hidden than their other YC peers, access to a piloted, psychosocial support programme.

In addition, we would argue that the pandemic brought with it an opportunity to make informal care in general, and care provided by YCs in particular, more visible and to bring YCs mental health and well-being to the attention of decision makers and policy makers, helping to remove the taboo around mental health. Further, it helped to challenge gender stereotypes in care roles.

### 4.4. Recommendations for Further Research and Action

Summing up, our core recommendations arising from the project directed at practitioners, decision makers and policy makers working with, coming into contact with YCs and AYCs or having a mandate to work for the issue of YCs include the following three messages:

(1) Identify YCs and AYCs: Young carers have remained in the blind spot of policy makers and practitioners for too long and they need to be paid attention to. The data arising from the ME-WE study cast light for the first time on many hidden aspects of adolescent caring roles. Further studies should investigate key aspects, such as AYCs risk of self-harm or harm to others, and more detailed studies of the nature of AYCs’ mental health problems and how these impact on their past and future education/university/workforce participation, aspirations, and independent lives.
(2) Recognise YCs as children in potential need of extra, tailored support: Young carers face a number of specific challenges as a result of their caregiving activities. They should therefore be approached as a group at risk and benefit from tailor-made policies and support measures.
(3) Listen to YCs: No policy or practice that impacts young carers should be developed without them. This principle builds on Article 12 of the UN Convention on the Rights of the Child (1989).

## 5. Conclusions

The main conclusions arising from the project are firstly that the project led to new substantive knowledge about AYCs, and the impact of caring on their mental health from a cross-national perspective, involving six European countries with a varied level of approaches and awareness of AYCs. Second, the active involvement of YCs and former YCs, together with various key stakeholders, throughout all phases of the project was a critical success factor in ensuring the responsiveness of the research, for example, the ME-WE model and programme and other intellectual outputs, and the knowledge translation activities. Third, in response to the COVID-19 pandemic, the psychosocial intervention was fully virtualised which enabled the running of the support programme in any circumstances, irrespective of geographical location. Fourth, the coordinated knowledge translation actions helped to put the issue of YCs on the policy agenda at national and European levels and led to several practice developments. In particular, the active engagement of Eurocarers, the pan-European association advocating for carers, in the consortium led to the project having a substantial impact on European stakeholders and policy makers, contributing to raising awareness and starting to include the issue in EU and national policy agendas. Finally, project partners in six European countries created (and will continue to create) close links with schools, health and social care services, civil society and policy makers, contributing to an increased awareness among relevant stakeholders of ways to identify, support and empower (A)YCs.

## Figures and Tables

**Table 1:** ME-WE Clinical Trial Study summary overview.

Design	Originally, a cluster-based randomised control trial (RCT) design with a two (arms) by three (times) repeated measures factorial design executed in six European countries (Italy, Slovenia, Sweden, Switzerland, the Netherlands, and United Kingdom). Cluster randomization was used to minimize contamination between intervention and control arms [30].
Recruitment channels	Recruitment of AYCs to the study was carried out in schools (SE, SI, CH) or geographical areas by the national project teams collaborating with relevant community-based service organisations and schools (NL, IT, UK). The country-based recruitment strategies included a range of recruitment methods, such as oral presentations in schools and youth centres, dissemination materials (brochures, posters), social media and traditional media and via referrals from health and social care professionals and school staff.Following the outbreak of the COVID-19 pandemic, in which schools were closed and face to face support services were postponed, recruitment strategies switched to social media campaigns (SE, CH) and to email and telephone communication with relevant stakeholders.
Inclusion criteria	(1) Being between 15 and 17 years of age; (2) Taking on caring tasks for family member(s) (e.g., parents, siblings, grandparents) or significant other (e.g., friend, schoolmate or neighbour) with a disability, chronic physical and/or mental health condition or substance use issue and/or problems related to old age [1,31].
Exclusion criteria	(1) Concurrently participating in psychotherapies or mindfulness-based interventions/programmes; (2) Having started a new psychotropic medication within the past 30 days or planning on starting or changing psychotropic medication during the course of the study; (3) Limited knowledge of the local language.
Assessment timeline	Both the ME-WE intervention and the waitlist control group were assessed at baseline (T0), immediately post-intervention for the ME-WE intervention group or after 7 weeks for the waitlist control group (T1), and at 3 months follow-up (T2).
Primary outcomes	- Psychological flexibility: Avoidance and fusion questionnaire for youth (AFQ-Y) [32]. - Mindfulness skills: Child and Adolescent Mindfulness Measure (CAMM) [33]. - Resilience: Brief Resilience Scale (BRS) [34]. - Subjective mental health: Warwick Edinburgh Mental Well-Being Scale (WEMWBS) [35]. - Quality of life: Kidscreen 10 [20]. - Subjective health complaints: HBSC Symptom Checklist (HBSC) [36]. - Caring-related quality of life: ad hoc questions. - Cognitive and emotional impact of caring: Positive and Negative Outcomes of Caring (PANOC) [18,19]. - Social support: Brief Social Support Questionnaire (BSSQ) [37].
Secondary outcomes	- Self-reported school, training or work experience, performance, and attendance: ad hoc items.
Post-Intervention Self Assessment	- Post Intervention Self-Assessment (PISA) [18], only for participants in the experimental groups; 7 dichotomous (Yes/No) items on the ME-WE intervention (e.g., “I enjoyed most of the activities”), 10 Likert-type items about changes related to participation (e.g., “I feel able to choose the level of care I provide”) and 5 open-ended questions.
Other country-dependent ad hoc questions	- Other country-dependent ad hoc questions on intervention satisfaction, comfort and experience.
COVID-19 delivery items	- Satisfaction with the online delivery of the ME-WE intervention; for the fully online intervention delivery, a Likert-type (0 = totally dissatisfied to 10 = totally satisfied) item was administered, plus 3 multiple-choice items on satisfaction with specific features and problems encountered; for all experimental groups, a 5-point Likert scale (ranging from always to not at all). - Three open-ended questions asked AYCs for the impact of the COVID-19 pandemic on their lives and mental and physical health, and whether they or their families were receiving the support and services they needed during the COVID-19 crisis. AYCs in the intervention group received a further open-ended question asking them how they experienced their participation in the ME-WE sessions during the pandemic, and the exercises and home activities proposed in the ME-WE intervention.

**Table 2:** Additional process evaluation data collection methods.

Ad-hoc online survey for stakeholders of the ME-WE project	It aimed at identifying and examining positive and negative experiences in the clinical trial study on the effectiveness of the ME-WE interventions for AYCs. The ad-hoc survey was designed by the Linnaeus University (LNU) team and the University of Ljubljana (UL) team with support of the ME-WE country partners. It consisted of both closed-end and open-ended questions on the success factors and challenges identified by stakeholders during the phases of recruitment and implementation of the ME-WE support intervention. Data were also collected to explore the influence of the COVID-19 pandemic on recruitment and implementation. Furthermore, the survey was designed to gather information on the impact of the ME-WE project on the work with, and the level of awareness of, AYCs amongst stakeholders. Demographic data concerning the respondents were collected (gender, year of birth, profession). The online survey could be completed using multiple devices (computer, tablet, mobile phone) with an anticipated completion time of around 5–7 min.
Qualitative focus group and/or individual interviews with stakeholders in all countries	An interview guide was provided by LNU covering four topics: information and recruitment, implementation, external factors and suggestions for the future. Informed consent was obtained for all participants who consisted of ME-WE stakeholders in each of the six partner countries. Background information on gender, age, level of highest education, job title, and years spent working with children and young people were collected for all the participants.

**Table 3:** Overview of respondent demographics across the six countries.

	Total Respondents	No. of 15–17 Year Old Respondents	No. of AYCs	No. of Male AYCs	No. of Female AYCs	No. of Transgender AYCs
Italy	981	893	214	67	141	1
Netherlands	719	630	199	48	141	3
Slovenia	1122	1013	342	34	298	1
Sweden	3414	3015	702	238	447	2
Switzerland	2343	871	240	45	193	0
UK	859	724	402	126	256	8
**Total**	**9437**	**7146**	**2099**	**558**	**1476**	**15**

**Table 4:** Number and proportion of recipients of care indicated by AYCs across the six countries (multiple answers possible per AYC in each country).

	Mother	Father	Grandmother	Grandfather	Sister	Brother	Friend	Partner
Italy	18 (12.9%)	15 (10.7%)	68 (48.6%)	34 (24.3%)	7 (5.0%)	13 (9.3%)	84 (81.6%)	7 (6.8%)
Netherlands	70 (44.6%)	40 (25.5%)	19 (12.1%)	12 (7.6%)	24 (15.3%)	46 (29.3%)	62 (83.8%)	9 (12.2%)
Slovenia	81 (32.9%)	72 (29.3%)	69 (28.0%)	45 (18.3%)	27 (11.0%)	30 (12.2%)	124 (74.3%)	24 (14.4%)
Sweden	190 (49.6%)	131 (34.2%)	18 (4.7%)	26 (6.8%)	79 (20.6%)	72 (18.8%)	403 (84.8%)	40 (8.4%)
Switzerland	51 (31.7%)	25 (15.5%)	42 (26.1%)	21 (13.0%)	20 (12.4%)	27 (16.8%)	77 (63.6%)	11 (9.1%)
UK	183 (54.3%)	79 (23.4%)	26 (7.7%)	16 (4.7%)	75 (22.3%)	85 (25.2%)	138 (82.6%)	24 (14.4%)
**Total**	**593 (41.6%)**	**362 (25.4%)**	**242 (17.0%)**	**154 (10.8%)**	**232 (16.3%)**	**273 (19.2%)**	**888 (80.2%)**	**115 (10.4%)**

Note: valid percentages are presented, ignoring missing values. Percentages reflect proportion of AYCs selecting each option among those who had indicated that they care for a family member (n = 1444) for the initial six columns, and those who indicated that they care for a close friend (n = 1121) for the final two columns.

**Table 5:** *T*-tests on mean MACA score (SD) for AYCs and 15–17-year-old non-carers, separately for each country.

	AYCs	Non AYCs	*T*	*Df*	*p*	*d*
Italy	11.42 (5.38)	8.33 (4.51)	7.54 *	307.83	<0.001	0.62
Netherlands	12.24 (5.37)	7.48 (3.58)	11.35 *	280.98	<0.001	1.04
Slovenia	14.22 (5.81)	10.81 (4.62)	9.35 *	555.99	<0.001	0.65
Sweden	10.92 (4.97)	8.50 (4.12)	11.46 *	964.42	<0.001	0.53
Switzerland	13.15 (5.84)	9.66 (5.96)	7.65	846	<0.001	0.59
UK	14.44 (5.72)	7.95 (4.12)	17.41 *	692.39	<0.001	1.30
**Total**	**12.57 (5.64)**	**8.81 (4.57)**	26.73 *	**3210.93**	**<0.001**	**0.73**

Note. * equal variances not assumed. Reprinted with permission from Ref. [8].

**Table 6:** *T*-tests on mean MACA score (SD) for male and female AYCs, separately for each country.

	Male AYCs	Female AYCs	*T*	*Df*	*p*	*d*
Italy	10.59 (5.27)	11.85 (5.41)	1.56	203	0.121	0.24
Netherlands	10.15 (4.16)	12.86 (5.22)	3.65 *	101	<0.001	0.57
Slovenia	12.59 (6.30)	14.46 (5.65)	1.81	323	0.072	0.31
Sweden	10.74 (5.32)	11.02 (4.79)	0.68	653	0.494	0.06
Switzerland	12.93 (6.40)	13.22 (5.75)	0.29	229	0.774	0.05
UK	11.98 (3.99)	15.64 (6.14)	6.91 *	372	<0.001	0.71
**Total**	**11.24 (5.16)**	**13.07 (5.70)**	6.80 *	**1051.11**	**<0.001**	**0.34**

Note. * equal variances not assumed.

**Table 7:** Formal and informal support received in connection to caring role, as indicated by AYCs across the six countries.

	Familial Adult Working and in Receipt of Wage	Family in Receipt of Government Assistance	AYC in Receipt of Support	Family in Receipt of Support	School Awareness of Caring	Employer Awareness of Caring	Friend Awareness of Caring
Italy	205 (97.6%)	50 (23.8%)	46 (22.1%)	58 (27.6%)	23 (10.8%)	10 (4.8%)	93 (44.1%)
Netherlands	169 (94.9%)	79 (45.9%)	39 (22.4%)	62 (35.8%)	52 (30.8%)	22 (13.1%)	107 (62.2%)
Slovenia	301 (97.1%)	67 (22%)	42 (13.8%)	91 (30.1%)	43 (14.2%)	13 (4.3%)	134 (44.5%)
Sweden	661 (96.1%)	186 (27.2%)	279 (41.8%)	77 (11.4%)	213 (31.8%)	31 (4.7%)	342 (51.3%)
Switzerland	210 (94.6%)	52 (24.0%)	37 (16.8%)	41 (18.8%)	20 (9.1%)	29 (13.5%)	140 (63.6%)
UK	267 (72.8%)	236 (64.5%)	168 (45.8%)	165 (46.2%)	215 (58.6%)	36 (10.1%)	247 (67.1%)
**Total**	**1813 (91.8%)**	**670 (34.4%)**	**611 (31.5%)**	**494 (25.5%)**	**566 (29.2%)**	**141 (7.4%)**	**1063 (54.8%)**

Note. The valid percentage is presented, ignoring missing values.

**Table 8:** Stakeholders’ professional fields and professions.

Professional Fields	Professions/Work Title
Social and health care	Youth counsellor Field secretary Family therapist Youth nurse Psychologist
Schools	Teacher Student coach School social worker School link worker
Youth centres	Youth worker Youth worker trainee
Higher education	Researcher Educator
Decision makers and advisors	Head of unit for individual and family care Youth coordinator Coordinator informal care Head of youth centre School developer Head of student health care Head of education Didactic coordinator NHS Manager Consultant Counsellor
Others	NGO worker Professional text writer Publisher Fundraiser Co-founder of a caring organisation Project manager Participation worker ME-WE intervention facilitator

**Table 9:** Session discussion topics, themes and outcomes.

	Focus	Key Themes
Years 1–2	Co-design of the intervention, including the mobile app	(a) Identify a suitable model for the ME-WE intervention; (b) Finalise the design of the ME-WE mobile app; (c) Identify recruitment strategies to be adapted and implemented in each country.
Year 3	Support to the field work	(a) Recruitment strategies, rewards and recognition for participating AYCs; (b) Amendments in recruitment strategies and implementation plans, due to national COVID-19-related lockdowns or restrictions concerning travels, physical meetings etc.; (c) How to overcome the challenges in recruitment and implementation due to the pandemic lockdowns/restrictions; (d) Online booklet for AYCs, content and development; (e) YCs of older family members, their caring activities, difficulties and needs; (f) How participating AYCs and facilitators experienced the ME-WE model; (g) Development of national sustainability plans for work post-project.
Year 4	Preliminary intervention study findings	

**Table 10:** Knowledge translation actions, activities and key outputs achieved.

Knowledge Translation Action	Activities	Outputs
Awareness raising	Production of 11 scientific publications, which have been published on open access journals (more planned and under development).	Nap, H.H; et al. The awareness, visibility and support for young carers across Europe: A Delphi study. *BMC Health Serv. Res.* 2020, *20*, 921. doi:10.1186/s12913-020-05780-8. [9] Santini, S.; et al. Positive and Negative Impacts of Caring among Adolescents Caring for Grandparents. Results from an Online Survey in Six European Countries and Implications for Future Research, Policy and Practice. *Int. J. Environ. Res. Public Health* 2020, *17*, 6593, doi:10.3390/ijerph17186593. [10] Berger, F.; Guggiari, E.; Wirth, A.; Phelps, D.; Leu, A. Die Sichtbarkeit und Unterstützung von Young Carers in der Schweiz. *Krankenpfl. –Soins Infirm.* 2020, *113*, 21–22. [45] D’Amen, B.; Socci, M.; Santini, S. Intergenerational caring: A systematic literature review on young and young adult caregivers of older people. *BMC Geriatr.* 2021, *21*, doi:10.1186/s12877-020-01976-z. [17] Leu, A.; et al. Cross-national Analysis of Legislation, Policy and Service Frameworks for Adolescent Young Carers in Europe. *J. Youth Stud.* 2021, 1–21, doi:10.1080/13676261.2021.1948514. [7] Casu, G.; Hlebec, V.; Boccaletti, L.; Bolko, I.; Manattini, A.; Hanson, E. Promoting Mental Health and Well-Being among Adolescent Young Carers in Europe: A Randomized Controlled Trial Protocol. *Int. J. Environ. Res. Public Health* 2021*, 18*, 2045, doi:10.3390/ijerph18042045. [16] Guggiari, E.; Wirth, A.; Leu, A. Young Carers in Europe. Erfahrungen aus einem internationalen Horizon 2020 Projekt. *Onkologiepflege* 2021, *1*, 40–41. [46] Phelps, D.; Guggiari, E.; Leu, A. Adolescent Young Carers erreichen und unterstützen. Über die Schwierigkeit, Jugendliche während der COVID-19-Pandemie zu erreichen. *Schweiz. Z. Für Heilpädagogik* 2021, *27*, 45–51, www.szh-csps.ch/z2021-12-06 (accessed on 12 July 2022). [47] Guggiari, E.; Phelps, D.; Leu, A. Rekrutierung von «Adolescent Young Carers» in der Schweiz. Erfahrungen aus dem internationalen Horizon2020 ME-WE-Projekt. *Krankenpfl. -Soins Infirm.* 2022, *115*, 36–37. [48] D’Amen, B.; Socci, M.; Di Rosa, M.; Casu, G.; Boccaletti, L.; Hanson, E.; Santini, S. Italian Adolescent Young Caregivers of Grandparents: Difficulties Experienced and Support Needed in Intergenerational Caregiving—Qualitative Findings from a European Union Funded Project. *Int. J. Environ. Res. Public Health* 2022, *19*, 103, doi:10.3390/ijerph19010103 [49] Lewis, F.; Becker, S.; Parkhouse, T.; Joseph, S.; Hlebec, V.; Mrzel, M.; Brolin, R.; Casu, G.; Boccaletti, L.; Santini, S.; et al. The first cross-national study of adolescent young carers aged 15–17 in six European countries. *Int. J. Care Caring* 2022, doi:10.1332/239788222X16455943560342 [8]
	Organisation and participation in numerous events to raise awareness on YCs and to share the project findings.	10,000 stakeholders reached at national/regional/local level and 3000 stakeholders at European level.
	Translation of research findings in layperson terms and conveyed to relevant stakeholders via non-scientific publications.	A series of Policy briefs, six country-specific (Italy, the Netherlands, Slovenia, Sweden, Switzerland and UK) and one European, conveying in layperson terms the research findings from WP1, 2 and 3 and identifying policy recommendations. Italy: http://me-we.eu/wp-content/uploads/2019/06/Me-We-policy-brief-Italy.pdf (accessed on 12 July 2022) The Netherlands: http://me-we.eu/wp-content/uploads/2019/06/Me-We-policy-brief-The-Netherlands.pdf (accessed on 12 July 2022) Slovenia: http://me-we.eu/wp-content/uploads/2019/06/Me-We-Policy-brief-Slovenia.pdf (accessed on 12 July 2022) Sweden: http://me-we.eu/wp-content/uploads/2019/09/Me-We-Policy-brief-Sweden.pdf (accessed on 12 July 2022) Switzerland: http://me-we.eu/wp-content/uploads/2019/06/Policy-Brief_Switzerland.pdf (accessed on 12 July 2022) United Kingdom: http://me-we.eu/wp-content/uploads/2019/06/Me-We-policy-brief-UK.pdf (accessed on 12 July 2022)
		The Manual ‘*My day only starts when I finish school-Multi-stakeholders’ actions to support Young Carers’*, investigating the concrete actions that can be undertaken by different stakeholders (e.g., policy makers, health and social care providers, school professionals, youth, care workers, the media and the general public) to identify, support and listen to YCs. http://me-we.eu/wp-content/uploads/2021/01/MeWe-Manual-for-stakeholders.pdf (accessed on 12 July 2022)
		The ME-WE booklet, created by YCs for YCs, containing testimonials and tips to enable YCs to take care of themselves (while caring for another person) and to achieve their goals in life. https://me-we.eu/booklet (accessed on 12 July 2022)
		A series of Briefs on methodology and evaluation, based on the findings of WP5 and WP6, six country-specific, one European: “*The ME-WE Model–A co-created and scientifically tested support programme for adolescent young carers*”. Italy: http://me-we.eu/wp-content/uploads/2021/09/IT-PB-ME-WE_v2.pdf (accessed on 12 July 2022) The Netherlands: http://me-we.eu/wp-content/uploads/2021/09/NL-PB-ME-WE_v3.pdf (accessed on 12 July 2022) Slovenia: http://me-we.eu/wp-content/uploads/2021/10/SL-PB-ME-WE_v3.pdf (accessed on 12 July 2022) Sweden: http://me-we.eu/wp-content/uploads/2021/07/SW-PB-ME-WE-modellen_v2.pdf (accessed on 12 July 2022) Switzerland: http://me-we.eu/wp-content/uploads/2021/09/CH-PB-ME-WE_v2.pdf (accessed on 12 July 2022) United Kingdom: http://me-we.eu/wp-content/uploads/2021/09/UK-PB-ME-WE_v3.pdf (accessed on 12 July 2022)
	Use of social media, sharing their messages on occasion of national days dedicated to informal carers, YCs, or on International Days (Children’s Day and Mental Health Day).	3,000,000 stakeholders have been reached.
	Use of traditional media to raise awareness about YCs among the general public and to disseminate the ME-WE findings.	16 interviews on national TV or radio were released, reaching out to millions of citizens and stakeholders.
	Use of new tools for sharing knowledge, good practices and experiences on YCs.	The ME-WE/young carers repository was created, an online Repository of Evidence on multidisciplinary approaches to support AYCs in Europe–available to all, populated with research, policies and laws and practices–acting as a hub where relevant stakeholders can access valuable information on YCs and get inspiration.
Engagement	Development of multi-disciplinary approaches involving AYCs themselves, health and social care professionals (e.g., psychologists, psychiatrists, social workers, nurses, youth workers, YCs workers) as well as education professionals (e.g., head teachers, teachers and other education employees).	More than 8000 stakeholders were reached through BLNs and other events and activities with YCs and other target groups.
Change of policies and practices	Promotion of changes in practices, as the ME-WE project enabled professionals to be more attentive to signals of a caring role; to offer them support or to refer them to support services available at local level; to talk about but also talk with AYCs.	
	Increased (self)identification of YCs, thanks to the increased awareness and to the empowerment of professionals.	
	New partnerships working to support and identify YCs (e.g., between schools and care support centres or between social services, health care, youth centres and schools), which are still ongoing and in progress within project countries.	
	The support offers for YCs were strengthened, as the ME-WE intervention is an ambitious and ground-breaking support programme that is easy to replicate.	
	Empowerment of YCs, via their active involvement in all the project activities. AYCs played an active role in the BLNs, in the design and implementation of the ME-WE intervention and in the dissemination activities.	
	Influence of policies, advocating for YCs to be on policy agendas at European level (see for instance EuCa’s response–on behalf of the ME-WE consortium–to the targeted consultation on the Child Guarantee and the open consultation on the new EU Strategy for the Rights of the Child: https://eurocarers.org/contribution-of-eurocarers-to-the-european-commission-consultation-on-the-child-guarantee-advocating-for-the-inclusion-of-young-carers/, accessed on 12 July 2022) and national level (for instance, in the Netherlands, the National Alliance Young Carers, of which project partner VIL is a member, lobbied to draw attention to YCs and include them in the political agenda of the Ministry of Health: https://www.jmzpro.nl/de-alliantie/, accessed on 12 July 2022).	
	Partners prepared and are continuing to update country sustainability plans for ensuring that the impact of the ME-WE project will endure in the future. Sustainability plans include actions for, among other things: networking and working with YCs and stakeholders, training new facilitators and interested organisations, conducting further projects and systematic follow-ups based on the ME-WE intervention, exploiting the ME-WE mobile app, advocating and lobbying for YCs’ rights with local and national policy makers.	

**Table 11:** EU policy developments and contributions by ME-WE.

Topic	EU Policy Developments	Contributions by ME-WE
Rights of the child	The biggest achievement is in relation to the latest policy developments on children’s rights at EU level: the EU Strategy on the Rights of the Child [50] and the European Child Guarantee [51]. The goal of the Child Guarantee is to break the cycle of poverty faced by millions of children. The focus is on children in need (children from precarious households; children with a migrant background; children in institutions; and children with disabilities). The Child Guarantee acknowledges that reinforced and targeted support has to be put in place to ensure that children in need have equal opportunities in enjoying their social rights. The Child Guarantee initiative will contribute to the implementation of article 11 of the European Pillar of Social Rights, which states that “children have the right to protection from poverty. Children from disadvantaged backgrounds have the right to specific measures to enhance equal opportunities” European Commission, p.19 [52].	The ME-WE Project partners responded to the EC consultations related to these developments and advocated for a clear inclusion of YCs in the category “children in need” (in the sub-group “children from precarious households”). Indeed, research evidence, including the ME-WE survey findings, shows that YCs are at risk of poverty and social exclusion: unless adequately supported, they may face extra challenges compared to their peers in accessing their right to education, health (including mental health), leisure activities and nutrition. The ME-WE project partners were aware that the Child Guarantee left to each Member State the possibility to identify “children in need”. Yet, as the awareness about their existence and their needs is still low across Europe, it is very likely for YCs not to be seen as a target group for intervention in the way that other at-risk groups (e.g., children with migrant backgrounds or with disabilities) are. If no explicit reference was made at EU level, YCs may have been targeted by the States where awareness and support already exist, whereas they would have continued to be invisible in other Member States and their support needs would have remained unmet. Hence, the importance of a clear reference to YCs at EU level.As a result of these advocacy efforts, the Council Recommendation establishing a European Child Guarantee includes, in the definition of children in precarious family situations, examples related to the phenomenon of young caring (living with a parent with disabilities; living in a household where there are mental health problems or long-term illness; living in a household where there is substance abuse) [51]. This is a first, important success, on which the ME-WE project partners will build their advocacy efforts at national level, for a correct implementation of the Recommendation.
Gender equality in caring	The distribution of care work is one of today’s most significant challenges for gender equality [53]. The unequal distribution of caring responsibilities between women and men over the lifecycle is one of the drivers of the gender employment, pay and pension gaps. Hence, closing the gender care gap is one of the objectives of the EU Gender Equality Strategy 2020–2025 [54]. Gender roles in caregiving start emerging at a very early age: girls, more often than boys, are the ones to take on the care of their relatives who are chronically ill or have disabilities, along with other household tasks [55]. “Besides adult family members, many children are involved in providing care to family members who are ill or have disabilities and this has a major impact on their quality of life, education and mental health”. [55].	As a result of the advocacy efforts led by Eurocarers on behalf of the ME-WE consortium, YCs are explicitly mentioned in the European Parliament Report “Care services in the EU for Improved Gender Equality” [56]. In detail, the EP calls on the Commission and the Member States to undertake research on the numbers of young carers and on the impact of this role on their well-being and livelihoods and, on the basis of this research, to provide support and address the specific needs of young carers, in cooperation with NGOs and educational establishments.
Mental health	There is a growing momentum in the EU agenda around the topic of mental health. People’s well-being is not only a value in itself; it is a principle at the heart of the European project, as enshrined by Article 3 of the Treaty on the European Union [57]. Mental health and well-being have also an impact on inclusion, growth and sustainability. Mental ill health costs the EU an estimated 3–4% of GDP, mainly through lost productivity. Mental disorders are a leading cause of early retirement and disability pensions. Therefore, there is a growing recognition about the need for mental health to be considered and addressed throughout the policy-making process at the European and national levels. This has become even more evident after the COVID-19 pandemic, as the virus, and the policy responses to it, have intensified mental health challenges.	The project consortium, led by Eurocarers, has created a successful collaboration with the European Network of Ombudspersons for Children and presented the ME-WE project at their annual conference. As a result, the ENOC statement on child mental health, adopted at the ENOC General Assembly in 2018, includes a clear reference to YCs (a recommendation to develop support programmes for young carers to enable them to better enhance and protect their mental health) [58].

**Table 12:** Advocacy and lobbying actions by ME-WE to influence national policy and practice.

Country	Contribution by ME-WE to National Policy and Practice
Sweden	In Sweden, the Swedish Family Care Competence Centre (Nka), of which ME-WE project partner Linnaeus University acts as the research partner, secured financial support from the National Board of Health and Welfare Sweden (NBHWS) for the rollout of the ME-WE Model. This work commenced in the Autumn 2021, by educating and supporting health and social care professionals, school staff and representatives from civil societies in the interested 290 municipalities and 21 regions across Sweden to implement the ME-WE intervention. The NBHWS also agreed to actively promote and disseminate the core ME-WE project results and to support further research studies relating to ME-WE in Sweden.Due to the country-specific research evidence gathered in the project, the NBHWS now recognises that approximately three quarters of all children as next of kin in Sweden are actually YCs, which in turn has facilitated the work of Nka to successfully lobby for their commissioned programme of work for the period 2021–2024 to target YCs, starting with the setting up of a national User Forum, consisting of YCs (several of whom participated in the ME-WE project), to advise the NBHWS and the Public Health Agency of Sweden.
Slovenia	In Slovenia, the project partner established contacts with all high schools and all student dormitories in the country, as well as with hospitals, addiction centres and organisations dealing with individuals with impairments and/or special needs. The collaboration with the NGO SONČEČ (the Cerebral Palsy Association of Slovenia), via its involvement as a member in the national BLN, has proved particularly fruitful also for the long term: the NGO is willing to implement the ME-WE intervention even after the end of the project, by incorporating it in their summer camp.
Switzerland	In Switzerland, the ME-WE project partner was successful in increasing awareness about YCs among professionals, by presenting the topic to students in nursing and healthcare professions, as well as by writing publications in professional journals (e.g., Krankenpflege, the largest journals for professional nursing in Switzerland), in national languages. As a result of the ME-WE project activities, a rich network of engaged and motivated organisations has been established and some stakeholders have changed their working practices in relation to identifying AYCs. For example, professionals in schools and hospitals have become more attentive to any signals of a caring role and now have this issue in mind. Furthermore, the difficulties with recruiting AYCs to the Swiss clinical trial study suggested that AYCs preferred an intervention which was less time consuming, thereby giving further support for the YCs’ “Get-togethers”, recognised by the Federal Office of Public Health as an example of best practice.
The Netherlands	In the Netherlands, the National Alliance Young Carer [59], of which ME-WE project partner Vilans is a member, lobbied to draw attention to young carers and include them in the political agenda of the Ministry of Health. This advocacy work resulted in young carers being part of the national social media informal care campaign entitled #Deeljezorg.Regional network meetings coordinated by a care support centre and a school that participated in the ME-WE research study facilitated discussing the topic of caregiving and ways to effectively support AYCs in a specific region. AYCs were also encouraged to participate actively in these network meetings. A handbook [60] on setting up regional meetings has been developed by the Dutch partners and made available online, so that other interested care support centres and schools can create regional partnerships. Vilans currently offers interested parties the possibility to participate in ME-WE train the trainer sessions. Two care support centres have started this process and will start to facilitate the ME-WE training for YCs in the summer 2022.
Italy	In Italy, ANS was invited to talk about young carers during the “Mental Health Week” 2021 promoted by the National Health Service of Modena; following this event and the awareness raised among staff and managers, the Service decided to publish a call to employ a psychologist specifically appointed to the topic of YCs.The Metropolitan City of Bologna, which was involved in the piloting phase, issued a call to set up a local network of professionals supporting informal carers.
UK	In the UK, a promising collaboration among schools and Carers Trust Network Partners was established. Schools in the south-east of England have remained committed to supporting Carers Trust Network Partners to identify AYCs who may benefit from their services.One Carers Trust Network Partner has been asked to join the Clinical Commissioning Group steering group focusing on the mental health of young people in schools in their area, as a result of their participation in the ME-WE project.A national Young Carers Alliance has been established bringing together service providers, policy makers, professionals, researchers, and young carers themselves, to work together to raise awareness of children, adolescent and young adult carers, and to lobby for more and better services and support for them.

## Data Availability

The ME-WE project reports, deliverables and outputs are publicly accessible in the project website: https://me-we.eu (accessed on 12 July 2022). Open research data have been published in connection to related published papers.
